# Influence of urban activity in modifying water parameters, concentration and uptake of heavy metals in *Typha latifolia* L. into a river that crosses an industrial city

**DOI:** 10.1186/s40201-015-0161-7

**Published:** 2015-01-25

**Authors:** Stefan-Adrian Strungaru, Mircea Nicoara, Oana Jitar, Gabriel Plavan

**Affiliations:** Department of Biology, “Alexandru Ioan Cuza” University of Iasi, Faculty of Biology, 700505 Iasi, Romania; Department of Environmental Engineering and Management, ”Gheorghe Asachi” Technical University of Iasi, 73, “Prof. Dr. D. Mangeron” Street, 700050 Iasi, Romania

**Keywords:** Bioremediation, Heavy metals, Environmental interactions, Metal uptake, *Typha latifolia*, Urban activities

## Abstract

**Background:**

Heavy metals like Cu, Cd, Pb, Ni, Co and Cr can naturally be found almost all over this planet in various amounts. Urban activities such as heavy metal industry, traffic and waste can rapidly increase the metal concentrations in a fresh water ecosystem.

**Methods:**

This study was done in natural conditions to capture as many aspects in heavy metals pollution and bioremediation of Nicolina River, Romania considered a stream model which is under anthropogenic pressure. Water, sediment and leaves samples of *Typha latifolia* L. were collected during October 2013 and analyzed in order to assess certain heavy metals (Cu, Cd, Pb, Ni, Co and Cr) from each sampling site using GF-HR-CS-AAS with platform. Heavy metals in significant concentrations in cattail samples were correlated with the water parameters to show the possibility to use the cattail leaves as indicators in heavy metals pollution with potential in bioremediation because they can be easily harvested in autumn and this species is spread worldwide.

**Results:**

The levels of metals concentrations in leaves were: Cu > Ni > Cr > Pb > Co knowing that copper is an essential element for plants. The sampling time was important to draw the river diagnosis for heavy metal pollution. The samples were collected, from river, after more than 60 days without rain same as a “human patient” prepared for blood test. Cobalt was considered the metal marker because it was an element with the lowest level of usage in the city. Compared with it only lead, cadmium and copper were used intensively in the industrial activities.

**Conclusions:**

*T. latifolia* L. can be use as an indicator for the health of the studied stream and it was noticed that the heavy metals were not accumulated, although the metal uptake was influenced by sediments and water parameters. The alkalinity of the studied river acts as an inhibitor in the bioremediation process of cattail for cadmium and copper. Lead was uptake by leaves and the water parameters influenced it but it wasn’t concentrated enough in leaves to propose this species in lead bioremediation process for Nicolina River.

## Background

Human activities have a high pressure against the environment with damages of all ecosystems. Each living being deserves a healthy and a clean environment to be borne, rise, to reproduce and die. The organisms react different when the concentration of chemical compounds is increasing in the environment, some of them tolerating very fast the changes but the others suffering so much because of this [1]. Food can be easy contaminated with toxic compounds and this problem can produce damage to human health if it is not well monitored [2]. A stream that crosses a city or a small urban area may negatively interact with the anthropogenic activities, with a significant amount of pollutants. Some of these pollutants are represented by heavy metals from the industrial activities [3], the illegal landfills, traffic and industrial wastewaters released into the environment, without a prior treatment.

Heavy metals can persist for a long time in plant and animal tissues, even if they are in small amounts in the environment [4]. The water chemistry has an important role in heavy metal absorption by organisms within aquatic environments and it can be easily influenced by urban activities [5]. The heavy metals pollution in a river can be analyzed for water, sediments and biota. The seston in a river is in a direct relationship with the rainwater that transports these compounds from atmosphere and terrestrial environment very fast [6]. Sediments can directly influence the heavy metal pollution if they are mixed by the water streams [7]. In the aquatic environments, some parameters like pH, redox potential, salinity, other metals and ionic bounds affect the metal absorption in organisms [8]. Biological absorption of heavy metals is a friendly environment technology with large applications in the future. The plants have the capacity in removing toxic metals from water; also some fungus species have high potential in this process [9].

*Typha latifolia* L. is a wide spread macrophyte that grows very fast in biomass [10], with a high capacity of absorption and accumulation of heavy metals from the environment [11-15]; thus can be used as a bioindicator of heavy metal pollution [16] and in bioremediation processes. *Typha* sp. has the capacity to degraded fast the organic pollutants [17], which is very important in the treatment of the domestic water.

The study was conducted on the analysis and interpretation of the connection between the change of water parameters (pH, salinity, conductivity, ORP, TDS) and heavy metals concentrations (Cu, Cd, Pb, Ni, Co, Cr) in water, sediments and upper parts of *Typha latifolia* L. from Nicolina River, along its course through Iasi City in different areas affected by urban activities like heavy industry. It was studied the capacity of this species in bioremediation of heavy metals and the role as bioindicator for health of studied environment.

## Materials and methods

### Sampling area description

**Figure 1:**
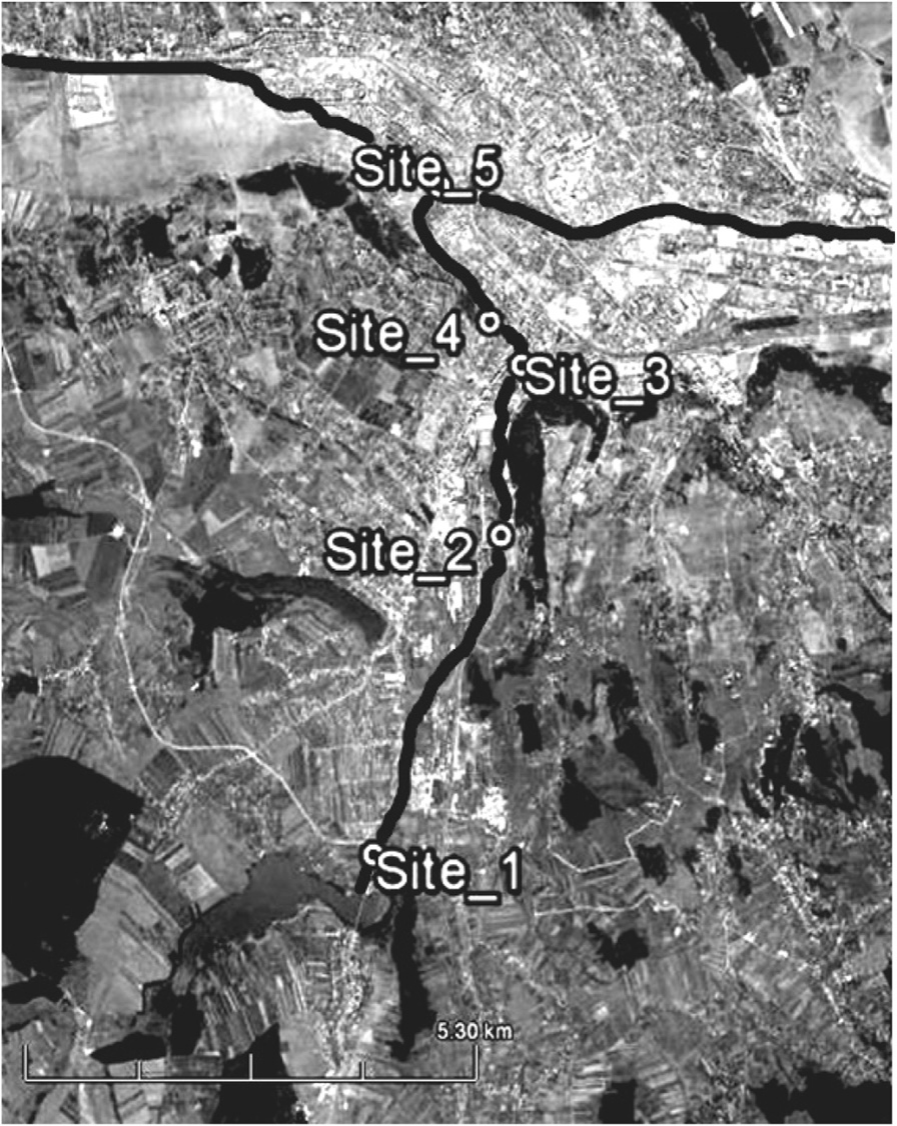
**Map of the sampling sites (Google Maps).**

Nicolina River crosses the Iasi City situated in the eastern side of Romania, along its way to Bahlui River, from the direction South to North. For this study five sampling sites (Figure 1) were selected and they were used as a model in this study. Site_1 is considered the reference sampling site and it is situated outside the city in Dumbrava village, an area without any industrial activities but with the traffic as the main pollution source. Site_2 is situated to the entrance in the city, close to the industrial area called in the past Heavy Equipment Works (CUG), at present days Fortus. Its activity was intensive between 1976 and 2003 period focused on construction of iron and steel heavy parts for nuclear reactors, cargo boats and others. Today only a small part is still active. Site_3 is situated 1 km downstream the industrial complex in a residential area with an intensive traffic. Site_4, located in a residential area and it is surrounded by vegetation and neighborhoods. Site_5 is situated 400 m upstream of the confluence with the Bahlui River. The sampling was conducted in October 2013 after more than 60 days with no rainfall.

The pH, salinity, conductivity, total dissolved solids (TDS) and redox potential (ORP) were measured at each sampling site in situ using a HI 9828 produced by Hanna Instruments, calibrated in the laboratory, 24 h before sampling. The period between the measurements at each sampling site was 20–30 minutes.

### Water samples preparation

The samples were collected from each sampling site in replicates (n = 3) in 250 ml polyethylene (PE) sterile bottles, prewashed with sampling water and acidified to a pH < 2 with HNO_3_ 65% Suprapur, Merck for preservation [18,19]. In the laboratory the samples were prepared for microwave mineralization in TFM pressure vessels, carefully mixing 25 ml of water sample with 1.5 ml HNO_3_ 65% Suprapur. The mixture needed 15 minutes to react before mineralization in 3 steps program of the MWS-2, Berghof following the protocol recommended by producer. After mineralization, the samples were kept in sterile 30 ml PE bottles for metal analysis.

### Sediment samples preparation

The sediments were collected in replicates (n = 3) from each sampling site, in a 20 cm column using a polycarbonate (PC) corer, 7 cm diameter. Each sample was stored in PE bags in a cooling box until the preparation for the next step. In laboratory they were slowly dried at 75°C, grinded in fine powder and sorted the small stones. The fine powder was stored in PE bags and shacked for 5 minutes. 0.5 g from each sample were weighted and mixed in TFM pressure vessels with 2 ml HCl 37%, 2 ml HNO_3_ 65% Suprapur and 1 ml H_2_O_2_ 30% Merck. The mixture needed 25 minutes to react before mineralization in 3 steps program for sediments. After mineralization, the samples were filtered using filter paper for quantitative analysis (ashless) IDL GMBH &CO in 100 ml flasks and washed with ultrapure water until it reached the 100 ml volume for each sample. After filtration and dilution, the samples were kept in the flasks for heavy metal analysis.

### *Typha latifolia* L. tissues samples preparation

The most important for study were the cattail leaves because they can be harvested every year in the same sampling period (October-November, 2013) and they may clean the environment by concentrating the heavy metals. This macrophyte was present in the highest biomass and abundance in the end of vegetation season. It was absent at Site_5. This species was identified in the field as *Typha latifolia* (L.) and they were sampled leaves in replicates (n = 3) from biomass above the water surface from each sampling site. The samples were stored in PE bags in a cooling box. In the laboratory, the samples were washed with ultrapure water, chopped with plastic tools, dried at 85°C and grinded. 0.15 g of each sample were weighted and mixed in TFM pressure vessels with 1 ml HCl 37%, 2 ml HNO_3_ 65% Suprapur and 0.5 ml H_2_O_2_ 30% Merck. The mixture needed 25 minutes to react before mineralization in 3 steps program for dried plants. After mineralization, the samples were filtered using filter paper for quantitative analysis (ashless) in 50 ml flasks and washed with ultrapure water until was reached a 50 ml volume. After filtration and dilution, the samples were kept in the flasks for metal analysis.

### Apparatus optimization for metal analysis and method validation

Metals measurement was performed using a GF-HR-CS-AAS with platform contrAA600, AnalytikJena. For each metal, the AAS was optimized and calibrated using stock solutions for Cu, Cd, Pb, Ni, Co and Cr diluted from standard certificated solutions 1000 mg L^−1^ (Merck) in ultrapure water with 0.05% HNO_3_. The samples needed Palladium matrix modifier. It was studied the signal and the spectrum of each sample to avoid the interferences and possible contaminations. During the measurements there were conducted QC (Quality Control) analyzes for each metal using certificated solutions. The linear calibration and the method validation results are presented in Tables 1, 2, 3, 4, 5 and 6 for each studied metal.

**Table 1:** **The calibration and quality control results for lead measurements**

**Pb (217.005 nm)**					
**Evaluation**					
**Line**	**Int. mode**	**BGC mode**	**Spectr.range**	**Eval.pixels**	**BGC fit**
Pb217	Area	with reference	200	5	dynam.
**Compute calibration**		**Quality control**			
R^2^(adj.): 0.997703286	**Sample**	**Nominal val.**	**Recovery**		
Method SD: 0.38421 μg/L	1	6 μg/l	105.7% OK		
Char.conc.: 0.39746 μg/L	2	6 μg/l	104.3% OK		
	3	6 μg/l	105.9% OK

**Table 2:** **The calibration and quality control results for copper measurements**

**Cu (324.754 nm)**					
**Evaluation**					
**Line**	**Int. mode**	**BGC mode**	**Spectr.range**	**Eval.pixels**	**BGC fit**
Cu324	Area	with reference	200	5	dynam.
**Compute calibration linear**		**Quality control**			
R^2^(adj.): 0.989369193	**Sample**	**Nominal val.**	**Recovery**		
Method SD: 1.83130 μg/L	1	20 μg/l	91.7% OK		
Char.conc.: 0.20960 μg/L	2	20 μg/l	95.8% OK		
	3	20 μg/l	103.2% OK

**Table 3:** **The calibration and quality control results for cadmium measurements**

**Cd (228.801 nm)**					
**Evaluation**					
**Line**	**Int. mode**	**BGC mode**	**Spectr.range**	**Eval.pixels**	**BGC fit**
Cd228	Area	with reference	200	5	dynam.
**Compute calibration linear**		**Quality control**			
R^2^(adj.): 0.989476072	**Sample**	**Nominal val.**	**Recovery**		
Method SD:0.19918 μg/L	1	3 μg/l	101.2% OK		
Char.conc.: 0.03882 μg/L	2	3 μg/l	97.4% OK		
	3	3 μg/l	97.2% OK

**Table 4:** **The calibration and quality control results for nickel measurements**

**Ni (232.003 nm)**					
**Evaluation**					
**Line**	**Int. mode**	**BGC mode**	**Spectr.range**	**Eval.pixels**	**BGC fit**
Ni232	Area	with reference	200	5	dynam.
**Compute calibration linear**		**Quality control**			
R^2^(adj.): 0.989669840	**Sample**	**Nominal val.**	**Recovery**		
Method SD:0.71148 μg/L	1	8 μg/l	105.3% OK		
Char.conc.: 1.93116 μg/L	2	8 μg/l	99.1% OK		
	3	8 μg/l	108.1% OK

**Table 5:** **The calibration and quality control results for chromium measurements**

**Cr (359.348 nm)**					
**Evaluation**					
**Line**	**Int. mode**	**BGC mode**	**Spectr.range**	**Eval.pixels**	**BGC fit**
Cr359	Area	with reference	200	5	dynam.
**Compute calibration linear**		**Quality control**			
R^2^(adj.): 0.996788129	**Sample**	**Nominal val.**	**Recovery**		
Method SD:0.33459 μg/L	1	5 μg/l	93.6% OK		
Char.conc.: 0.43403 μg/L	2	5 μg/l	93.8% OK		
	3	5 μg/l	103.7% OK

**Table 6:** **The calibration and quality control results for cobalt measurements**

**Co (240.725 nm)**					
**Evaluation**					
**Line**	**Int. mode**	**BGC mode**	**Spectr.range**	**Eval.pixels**	**BGC fit**
Co240	Area	with reference	200	5	dynam.
**Compute calibration linear**		**Quality control**			
R^2^(adj.): 0.999345714	**Sample**	**Nominal val.**	**Recovery**		
Method SD: 0.15798 μg/L	1	4 μg/l	106.8% OK		
Char.conc.: 0.26280 μg/L	2	4 μg/l	104.3% OK		
	3	4 μg/l	104.7% OK		

### Metal concentration factor

The heavy metal concentration factor was calculated according to formula: CF = [M]_plant_/[M]_environment_ [20], where M is the metal concentration. This factor had been calculated as the report between leaf and sediment concentrations at each sampling site. Its value expressed metal’s bioconcentration and level of the pollution.

### Statistical analysis

For Normality Test it was performed the Shapiro-Wilk test. Statistical analyses for sediment, biota and *Typha latifolia* L. were performed using the One-Way ANOVA, followed by Tukey HSD test, in order to explain the influence of urban activities on river course. Pearson correlation followed by 2-tailed test of significance was performed between heavy metal from environment and cattail, water parameters and the significant metal concentrated in the cattail samples from all sampling sites. All the statistical analyses were carried out using OriginPro 8 software.

## Results

### Water analysis

**Figure 2:**
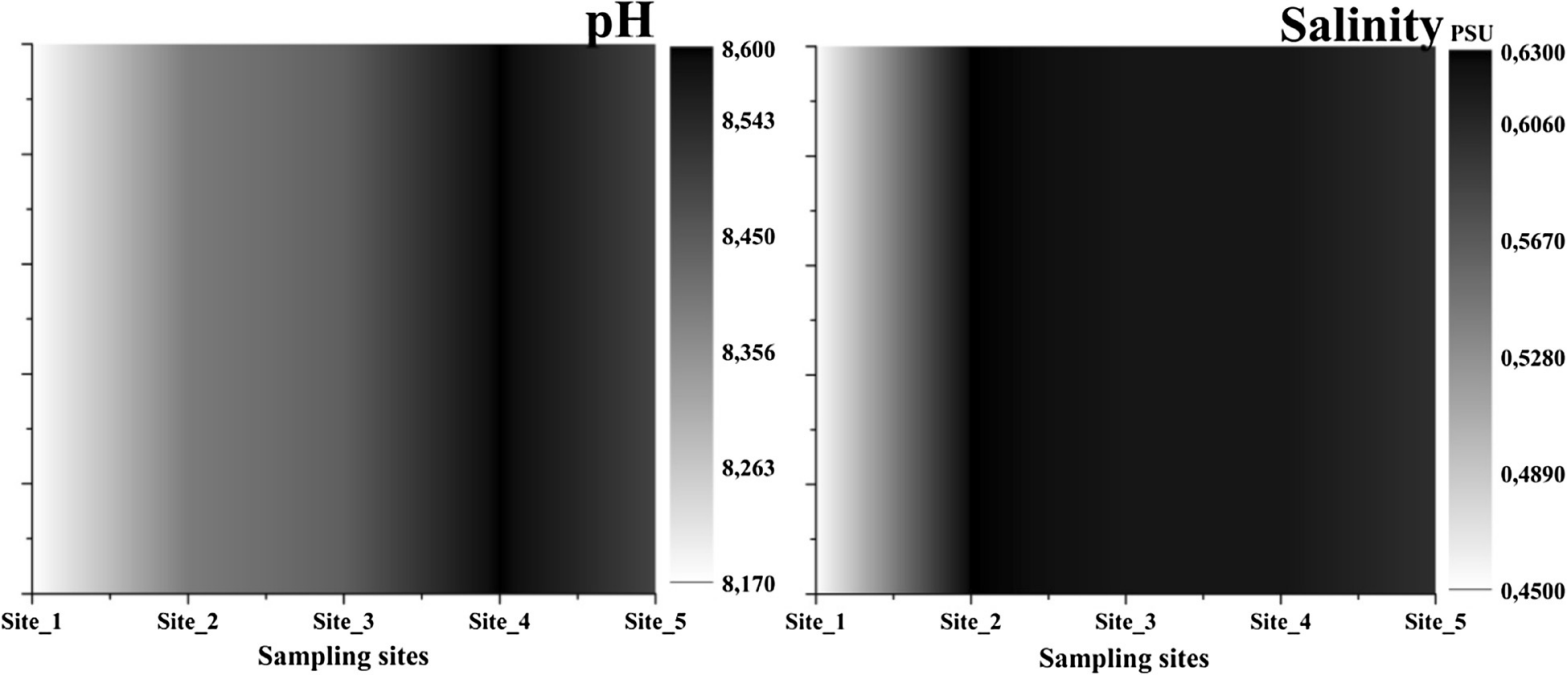
**Variations of pH and salinity in water.**

**Figure 3:**
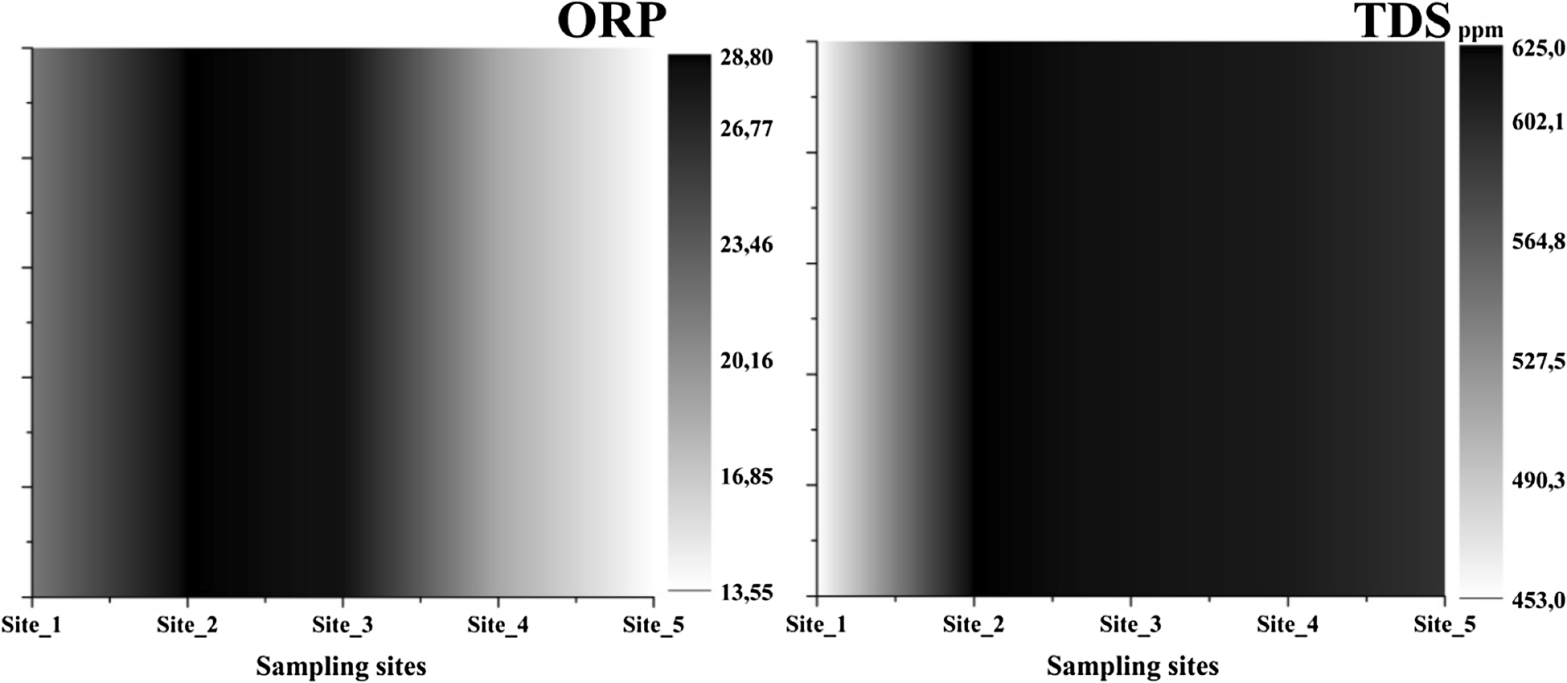
**Variations of ORP and TDS in water.**

**Figure 4:**
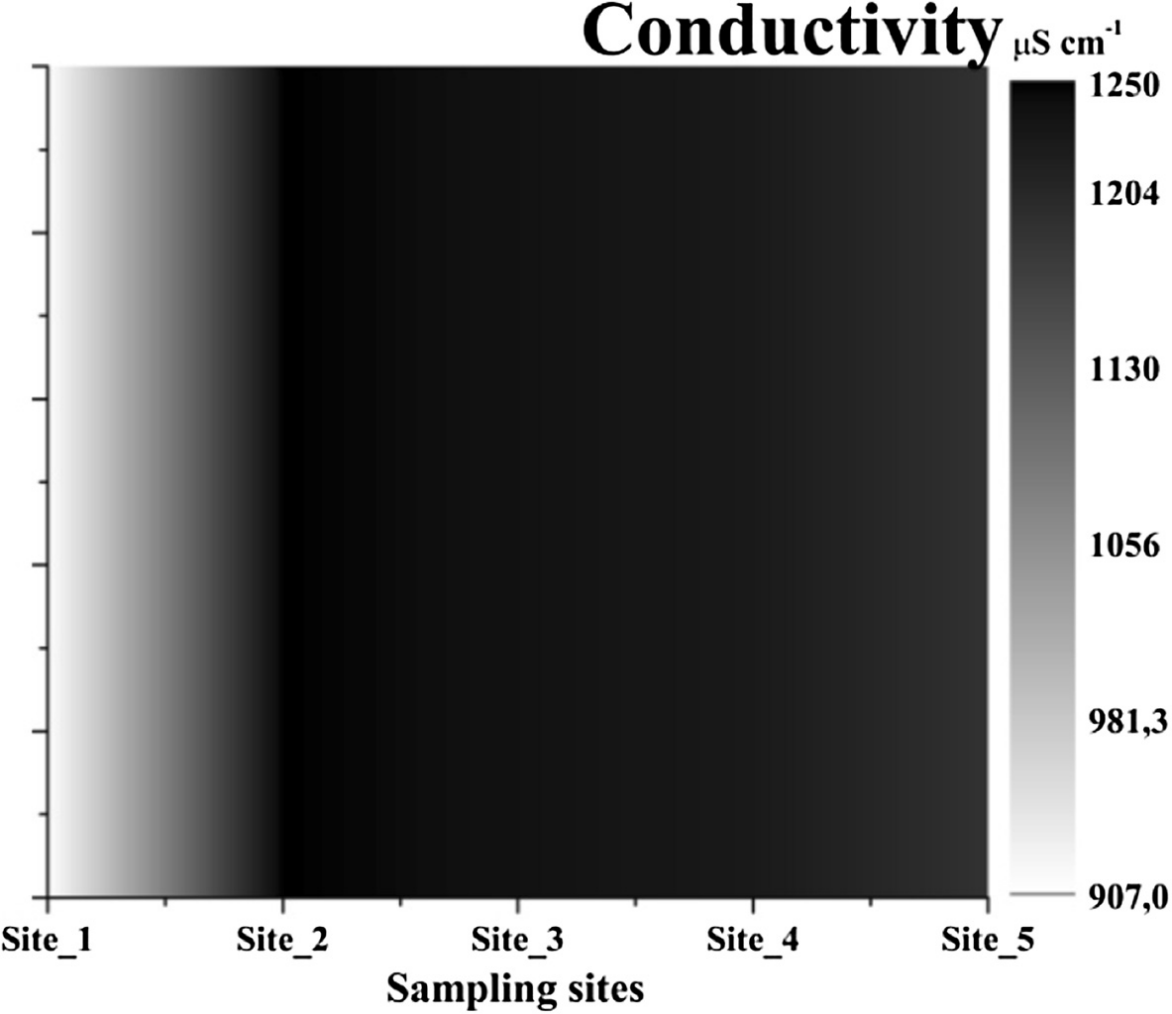
**Variations of conductivity in water.**

Water parameters analysed provided the first image about the influence of the city. The values of pH (Figure 2) were between 8.17-8.6 different for each sampling site which is characteristic for this area rich in limestone. The lowest value was recorded outside the city, at Site_1 while in the city the pH easily increased with the highest value at Site_4 - area with fine sediments and stones. There was recorded an increasing pattern for salinity 0.45-0.63 PSU, total dissolved solids (TDS) 453-625 ppm (Figure 3) and conductivity 907-1250 μS cm^−1^ (Figure 4) that suggested a strong variation of these starting from Site_2 near industrial area. The redox potential (ORP) was 13.55-28.8 with the highest value between Site_2 and Site_4, around the Heavy Equipment Works (CUG), at present Fortus, that appeared to have the highest influence in variation of this parameters. The lowest value was recorded at Site_5, which suggested once more how urban activities can fast change some parameters of the water.

**Figure 5:**
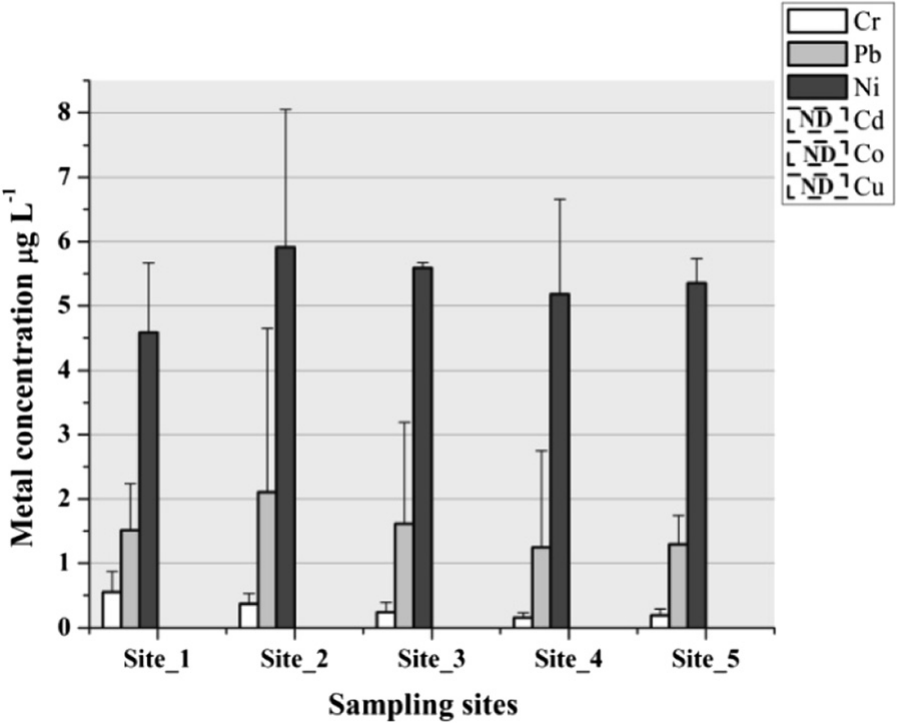
**Metal concentrations in water samples (ND-not detectable).**

There were identified and measured in water samples Cr, Pb and Ni (Figure 5). The other analyzed metals (Cu, Cd, Co) were under detection limit of the GF-HR-CS-AAS. The metal that was present in the highest concentration was nickel followed by lead and chromium. Nickel concentration did not exceed significant according to ANOVA (p > 0.05, df = 4, F = 1.61) even the averages were between 4.47-5.9 μg L^−1^. This result is explained by the absence of an active pollution source with Ni at present. Same explanation was in case of lead (p > 0.05, df = 4, F = 0.91) 1.28-2.1 μg L^−1^ but the chromium concentration in water had a significant difference (**p < 0.01, df = 4, F = 8.89) with averages 0.11-0.54 μg L^−1^. The highest values were recorded at Site_1, outside the city, in the area with the lowest anthropogenic activity; the water pH may influence the concentration of this metal, even if its value was closer to 8.

### Cattail analysis

**Figure 6:**
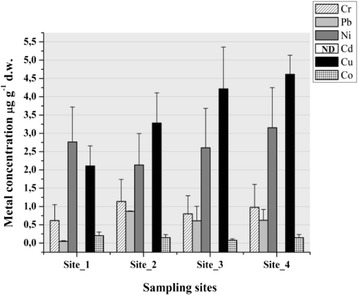
**Metal concentrations in leaves from** ***T. latifolia*** **(L.) d.w.-dry weight (ND-not detectable).**

In samples of *T. latifolia* (L.) tissues, the levels of cadmium were not detectable, but the others analyzed metals were quantified (Figure 6) in this order of the concentration: Cu > Ni > Cr > Pb > Co. Copper concentrations in samples were 1.67-7.17 μg g^−1^ (d.w., p > 0.05, df = 3, F = 1.86) with no significant variations between the sampling sites. There were not any significant variations for Ni 1.74-4.4 μg g^−1^ (d.w., p > 0.05, df = 3, F = 3.03), Cr 0.26-1.47 μg g^−1^(d.w., df = 3, F = 1.28) and Co 0.04-0.35 μg g^−1^ (d.w., p > 0.05, df = 3, F = 0.75). The only significant difference (p < 0.001, df = 3, F = 29.49) was for Pb 0.062-0.86 μg g^−1^ (d.w.).

### Sediment analysis

**Figure 7:**
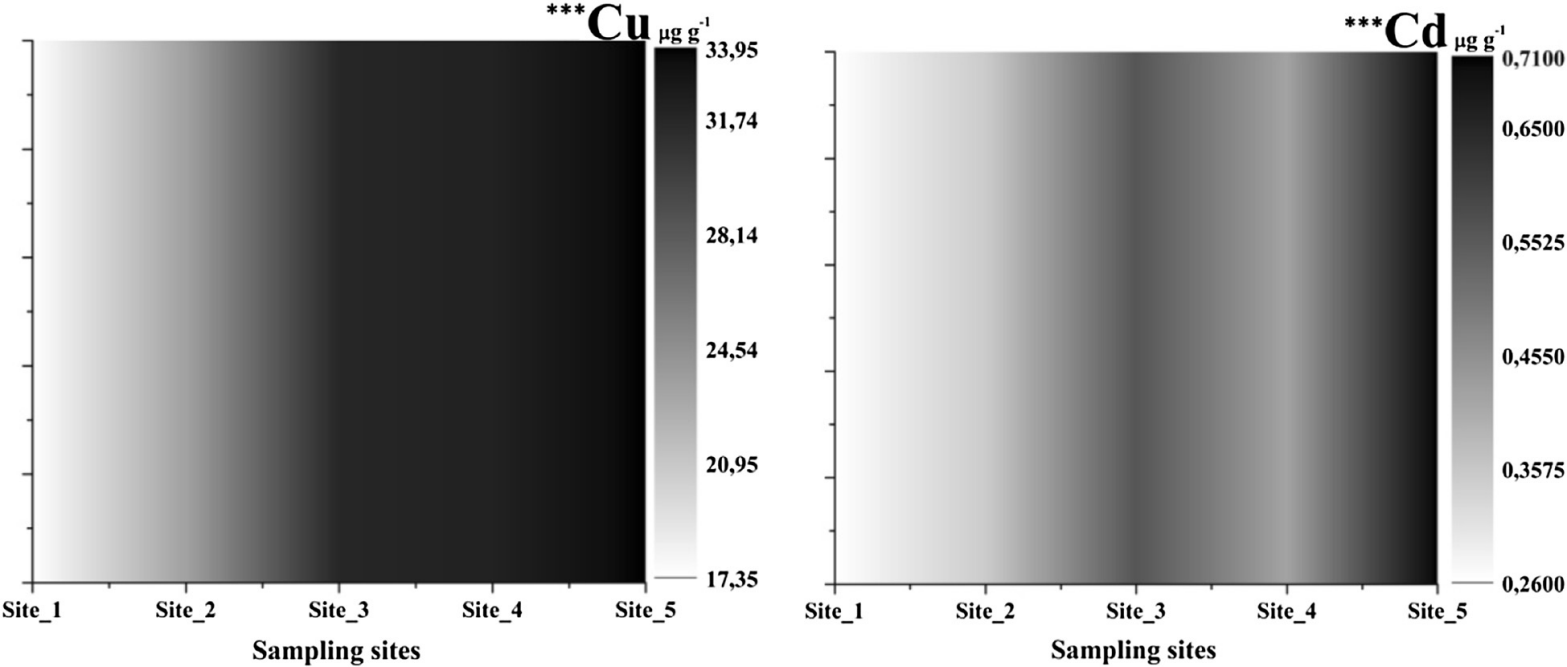
Copper and cadmium concentrations in sediment samples (***- p < 0.001, ANOVA Test of variance).

The highest variation of the concentrations was in the sediments samples. Copper (17.63–33.95 μg g^−1^, p < 0.001, df = 4, F = 48.05) increased from Site_2 in the industrial area and concentrated to Site_4 and Site_5. The concentrations for cadmium (0.26–0.71 μg g^−1^, p < 0.001, df = 4, F = 225.19) increased from Site_1 (Figure 7) to Site_5 where was the highest concentration. At Site_4 there was recorded a decrease in cadmium because of the sediment mixed with stones.

**Figure 8:**
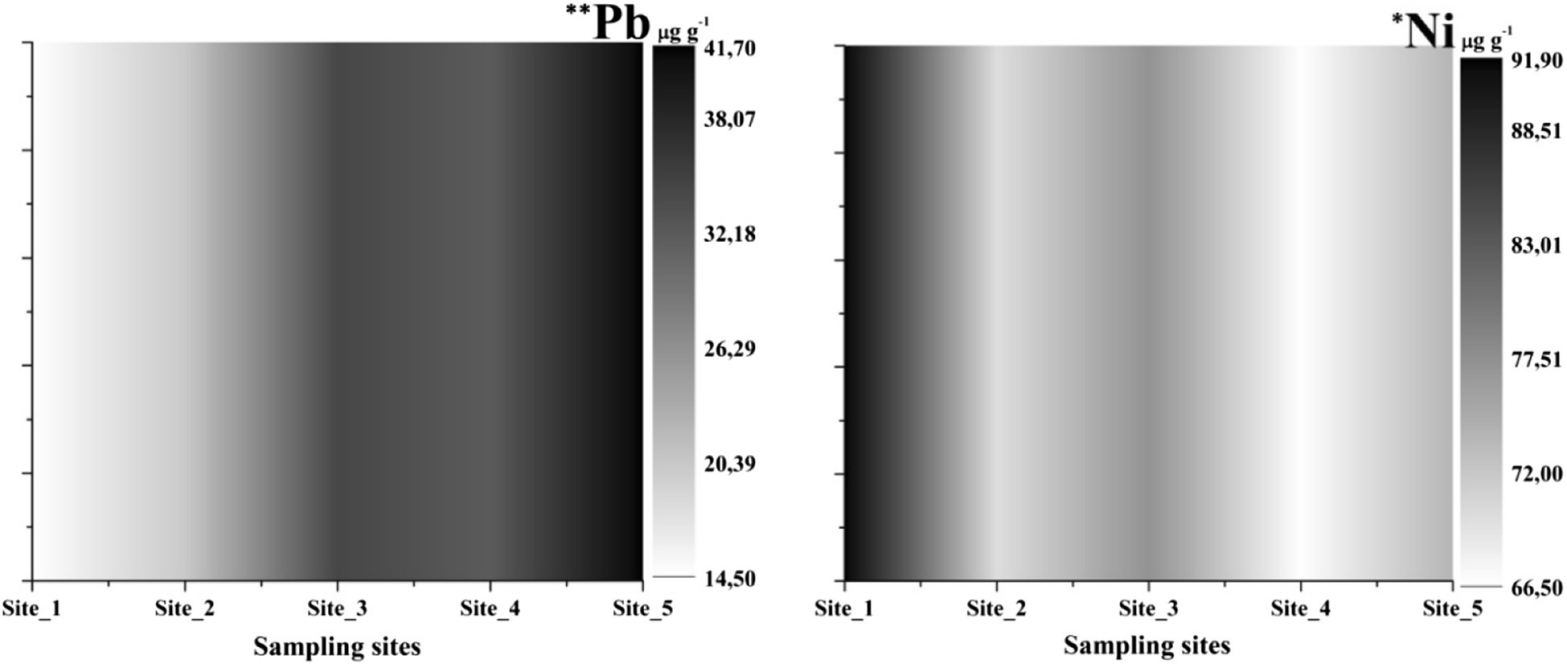
Lead and nickel concentrations in sediment samples (***- p < 0.001 and * - p < 0.05, ANOVA Test of variance).

The lead (14.50–41.70 μg g^−1^, p < 0.01, df = 4, F = 9.3) was concentrated at Site_5 with a slow decrease at Site_4. Downstream of the industrial area there was a significant increase of lead (Figure 8), possibly caused by the intensive traffic as well. In case of nickel (66.5–91.9 μg g^−1^, p < 0.01, df = 4, F = 4.35), the highest concentration was at Site_1 outside the city compared to the sites inside the city. For this metal, the city’s activities had no influence. The concentration with the lowest significant value was at Site_4 the area with the most stones in the substrate.

**Figure 9:**
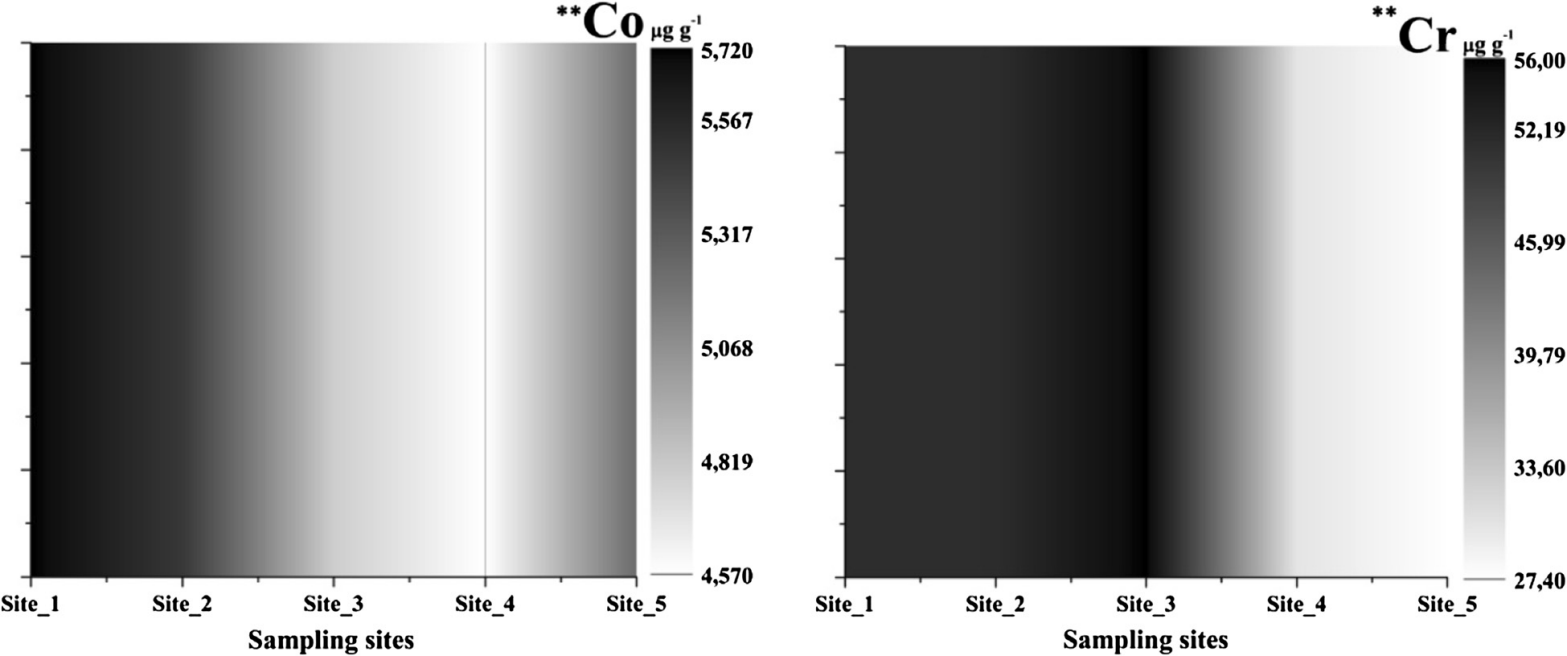
Cobalt and chromium concentrations in sediment samples (**- p < 0.01, ANOVA Test of variance).

Values for cobalt (4.57–5.72 μg g^−1^, p < 0.01, df = 4, F = 6.86) were highest in the samples outside (Figure 9) the city and they were not increased at the rest of the sampling stations; Site_4 had the lowest amount because of the substrate. Chromium (27.4–56 μg g^−1^, p < 0.01, F = 8.68) was the only analyzed element with high levels in samples both outside and inside the city.

## Discussions

### Heavy metal concentration factor

Present study used as a bioindicator for heavy metal pollution *T. latifolia* L. leaves. The level of pollution and transfer capacity were expressed by calculating the metal concentration factor. Sasmaz et al. [13] used the same report to express the metal transfer factor. This factor indicated low capacity of heavy metal absorption in this macrophyte in our study (Table 7). Heavy metal concentration in leaves tissues may depends on multiple variables, like water parameters. Their influence was well studied in toxicological laboratory experiments. The results provided the understanding of the mechanism of toxicity in case of heavy metals from aquatic solutions. The problem in this type of experiment is the reduction of the variables. Between the variable they exists interactions: environment - organism, organism-organism and inside the organism. The studied sites from the Nicolina River have different parameters for water, metal content from sediment and water. They were correlated with the uptake of the studied metals from environment. Copper is an essential micronutrient for plants and it has important role physiological functions of the plant [21]. In this study the copper was uptake in leaves in the highest concentration. This element increased in concentration from Site_1 to Site_4 for sediments but it had not significant differences in cattail leaves.

In Sasmaz et al. [13] the transfer factor was highest for cadmium and lead. In the present study, copper and nickel (considered to be another important micronutrient for plants) had the greatest values and the lowest one was for cobalt, chromium and lead (Table 7).

**Table 7:** **Metal concentration factor (MCF) in leaves samples from** *T. latifolia* **L.**

**Cu**	**Cd**	**Pb**	**Ni**	**Co**	**Cr**	**References**
0.77	1.070	0.91	0.74	0.51	0.35	[13]
(0.38-1.47)	(0.3-2.38)	(0.26-2.25)	(0.31-2.10)	(0.18-1.20)	(0.17-0.72)	
0.12	ND	0.018	0.035	0.027	0.02	Present study
(0.103-0.14)		(0.003-0.017)	(0.03-0.047)	(0.015-0.035)	(0.011-0.032)	

ND-not detectable.

MCF was expressed as average of the values interval.

### Heavy metal uptake from water in leaves

**Figure 10:**
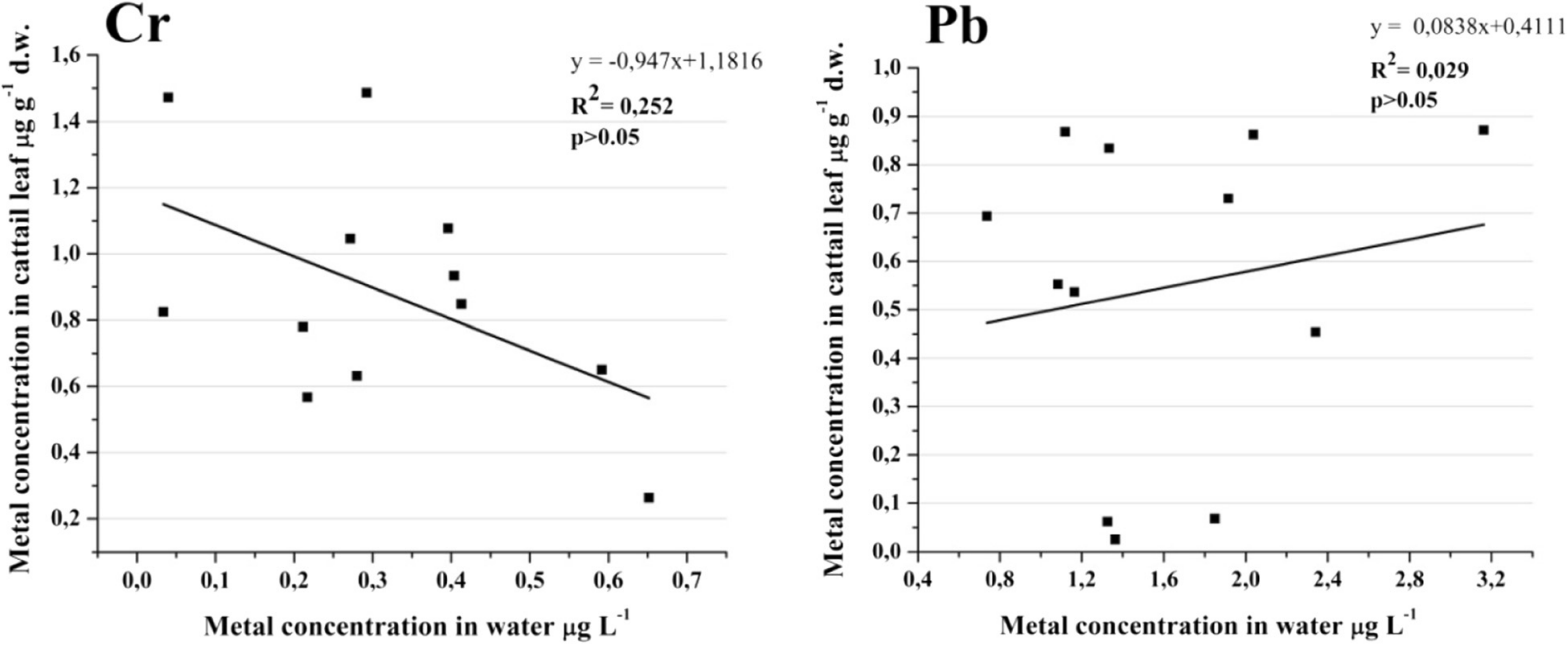
**Pearson correlation followed by 2-tailled test for chromium and lead.**

Chromium was the only heavy metal from water samples with a significant variation over the studied sites. It was applied the first correlation between the chromium from water and from cattail leaves. It was necessary to demonstrate and to show the uptake capacity of chromium from water by this macrophyte. It had an insignificant negative correlation between water and analyzed biota (Figure 10). In this case chromium from the water was not significantly uptake even if the concentration was higher for this aquatic macrophyte. Lead was the only metal with different concentrations in leaves from Site_1 to Site_2, but with no significance in water samples. We correlated its concentrations between water and cattail leaves. The lead from water was not the main source for cattail leaves. There was no significant correlation to show chromium and lead uptake by this macrophyte from water.

### Heavy metal uptake from sediments in leaves

**Figure 11:**
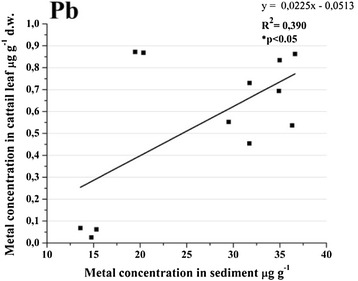
**Pearson correlation followed by 2-tailled test for lead in sediment and biota.**

The main source of lead in cattail samples was not from water. Sediments were the main source of lead in leaves samples and this was revealed by the positive correlation between sediments and leaves concentrations which were different for each sampling site (Figure 11). It uptakes the lead from sediments by roots to leaves. According to the heavy metal concentration factor, this macrophyte did not accumulated lead in leaves (MCF < 1), but the sediments enrichment with lead increased the uptake. The macrophytes from the urban area have more lead in significant concentrations than those located outside the city. Bioremediation processes are necessary inside the city.

### Water parameters and heavy metal uptake in biota samples

**Figure 12:**
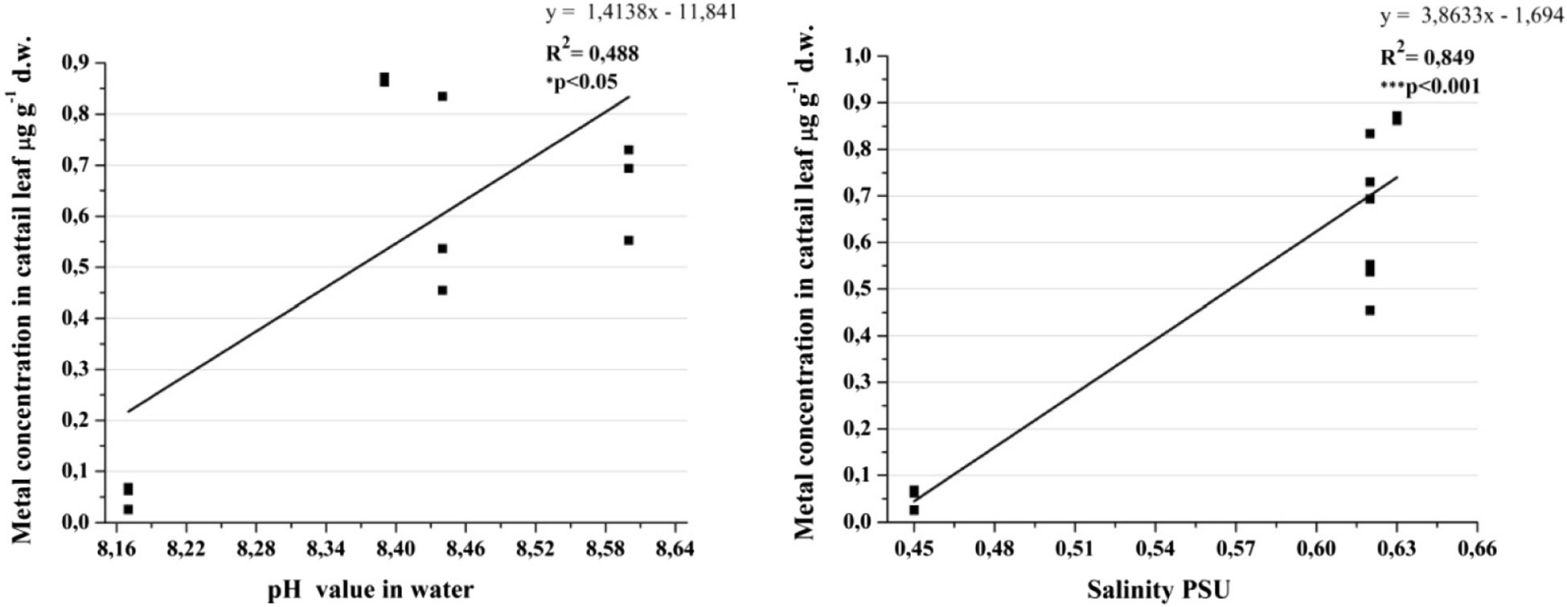
**Pearson correlation followed by 2-tailled test between Pb with pH and salinity.**

The water parameters values were different at each sampling site. Prasad et al. [8] explained the importance of these in heavy metal uptake from the environment by macrophytes. The present study showed, using the correlations, the influence of water parameters in the lead uptake by cattail leaves. For the first two parameters (pH and salinity) it was a strong positive correlation. Acidic medium increases the metals uptake into the plants parts [7]. In our study, an increasing of alkalinity was correlated with a higher amount of lead in cattail leaves (Figure 12). This can be possible as an adaptation of this species to an alkaline environment. The salinity was very strongly positively correlated with lead from the sample. The importance of salinity in metal uptake was well studied. Furthermore, it is possible that same species to uptake differently the metal at same salinity because of the interaction between other factors (physical, chemical, biological).

**Figure 13:**
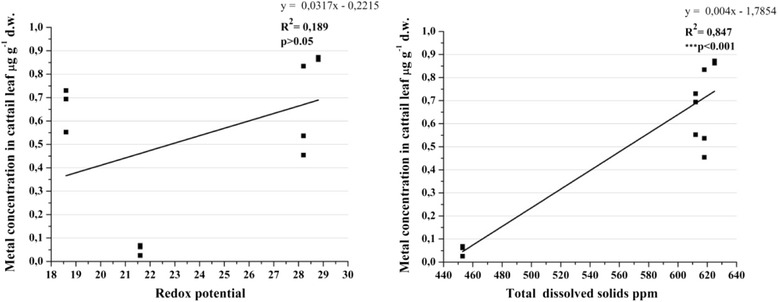
**Pearson correlation followed by 2-tailled test between Pb with Redox potential and total dissolved solids.**

The redox potential represents an important factor in metal uptake from sediments. In our study, this parameter had not a significant role (Figure 13) in metal uptake from sediments to leaves. It had the highest value between Site_2 and Site_3, the industrial area and it did not influence the metal uptake by leaves. The values were positive typical for an oxidative environment. Total dissolved solids (TDS) had a strong influence in metal uptake from sediments. It is possible that other compounds dissolved in water body (calcium, magnesium, carbonates, chloride) to increase the metal absorption capacity in leaves biomass even this did not accumulate the metal.

**Figure 14:**
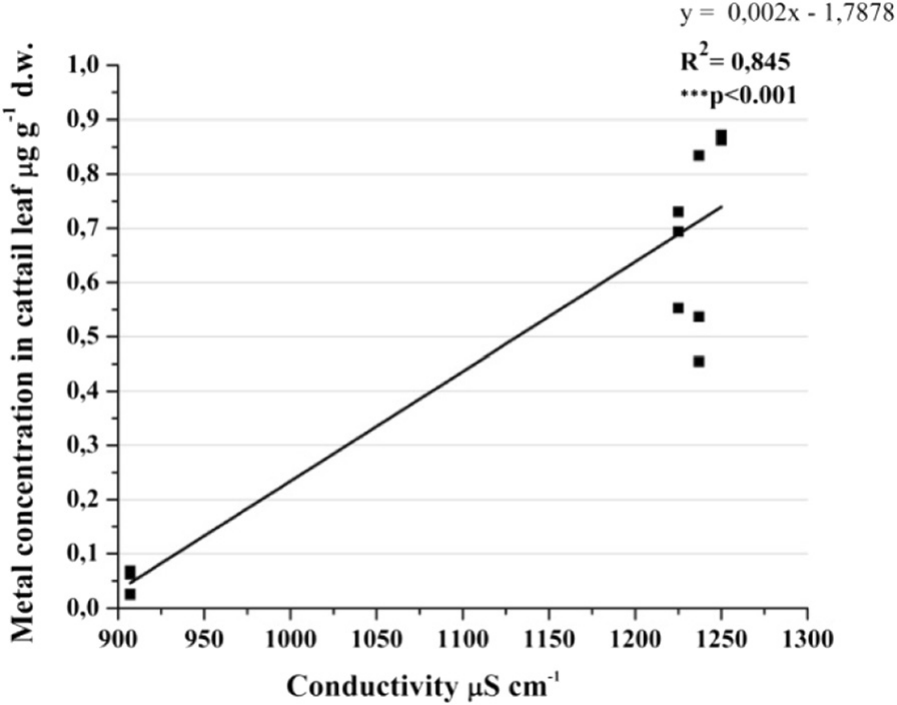
**Pearson correlation followed by 2-tailled test between Pb with conductivity.**

The connection between conductivity and lead in cattail leaf had a strong positive correlation that made the city activity to affect even more the lead uptake from the environment (Figure 14). Though there was a strong relation of heavy metal through biota-sediment-water parameters, the alkalinity of Nicolina River determinates a possible relative inactivation, being less harmful for the plants, concentrating into the sediments. The urban activities had an important role in uptake of the lead by *T. latifolia* L. They modified the water parameters and increased the capacity to absorb the metal from environment into the leaves.

## Conclusions

Urban activities from Iasi City had a direct impact upon water parameters from Nicolina River. These activities like steel and iron industry from the Heavy Equipment Works (CUG), at present Fortus, traffic and wastes increased the salinity, conductivity, TDS, pH and redox potential in the water body. There was not recorded a significant increase of the lead and nickel in water body inside the city, but a significant decrease of the chromium inside the city and possible of other different parameters was noticed. *T. latifolia* L. were used as a bioindicator for the health of this ecosystem and it was noticed that the heavy metals were not accumulated, although the metal uptake was influenced by sediments and water parameters. An increase of Cd, Pb and Cu in the city area was recorded for sediments and a decrease of Co, Cr and Ni. The water alkalinity, combined with other factors can reduce the toxic activity of these metals. *T. latifolia* L. cannot be use in the process of bioremediation in Nicolina River because it didn’t accumulated the studied metals.

This study represents a beginning in understanding the action of anthropogenic activities upon the environment at different levels and factors. These interactions will provide new data for future bioremediation experiments and in understanding of the chemical behavior (heavy metals uptake) of different species. There are many of variables in a stream that are influencing the organism’s behavior. To understand all the processes the measurement technology must to develop more and more.
